# Perceptions of Smartphone App Use among Mothers Raising Young Children

**DOI:** 10.3390/ijerph19137585

**Published:** 2022-06-21

**Authors:** Keum-Hee Jang, Song-Yi Lee

**Affiliations:** 1 Counselling and Coaching Department, Graduate School, Dongguk University, Seoul 04620, Korea; jjangmonica@naver.com; 2 Dharma College, Dongguk University, Seoul 04620, Korea

**Keywords:** mother, smartphone, Q-methodology, perception

## Abstract

The purpose of this study was to categorize subjective perceptions of smartphone app use among mothers with young children, derive characteristics of each perception type, and interpret the findings to determine appropriate smartphone use for each perception type. We applied the Q methodology to classify and analyze the types of perceptions of mothers with young children. Ultimately, we selected 40 Q samples by comprehensively analyzing in-depth interviews with mothers of young children and conducting reviews of literature related to smartphone app use. The P samples in the study consisted of 31 mothers. We performed data analysis using the QUANL program. The analysis identified four types of smartphone users: (1) “multi-adapters” who actively utilize functions, (2) “chaos dilemma” users who understand the disadvantages of smartphones but cannot refrain from using smartphone apps, (3) “time-killer dependent” users who utilize apps to relieve temporary stress and anxiety, and (4) “self-development focused users” who pursue positive changes. Based on these findings, we suggest ways in which mothers of young children can utilize smartphone apps in a developmental and appropriate manner.

## 1. Introduction

With the extensive innovation in information and communication technology, smartphones have become an essential medium in our daily lives [1]. All age groups around the world use smartphones because of their high mobility, accessibility, and few physical restrictions [2].

Smartphones were first introduced in Korea in 2009, a few years after their introduction in the US and the UK. However, the expansion of smartphone use in Korea was especially rapid [3]. Among smartphones’ various uses, network use has been expanded, and the number of related activities attracting interest and attention has increased [4]. The proliferation of smartphones has been accompanied by discussions about negative and positive aspects of their use. However, because smartphone use is so deeply ingrained in daily life, it is difficult to consider smartphones as simply negative or positive [5], as the perception may vary depending on the individuals who use them. Research has shown that the degree of smartphone use varies according to individuals’ personality traits [6], and the use of smartphones as social capital may also vary depending on personality (e.g., extraversion and neuroticism) [7]. Similarly, Richardson et al. [8] found four different types of motivation to use dating apps. This suggests that attitudes toward smartphone use can also differ depending on each person’s personality and characteristics.

For mothers who mainly stay at home raising their children, their use of smartphone apps satisfies their desire to obtain various information [9]. Additionally, because different content and services can easily be downloaded via smartphones, lifestyles and cultures have also changed [10]. Mothers use smartphone apps to manage risks and signs of disease at the time of pregnancy, which also provide a means for integrating data between healthcare providers and mothers, including dissemination of healthcare information and education, early detection of potential problems, and notification of critical events [11]. Expectant mothers use smartphones to obtain information related to pregnancy, childbirth, and childrearing [10]. Mothers devote a large portion of their daily schedules to care-related tasks, and they are often under great pressure due to a lack of sufficient parenting knowledge and a lack of opportunities for economic activity and social participation [12,13]. Regardless of their employment status, they become the primary performer of household chores and childrearing, and they sometimes feel restricted in their use of time and space. In such situations, smartphones with high portability, low cost, and easy access to wireless internet are an important medium for childrearing information [14].

Mothers also use smartphone apps for information, interaction, medical tools, and social media [15,16]. For example, Larkin et al. [14] found that mothers’ use of an app called Baby Mind to appropriately respond to their infants’ mental states demonstrates the efficiency of app use in childrearing. Additionally, mothers living in rural areas reported that such apps are convenient, as these apps taught them how to breastfeed [17]. Mothers also utilize smartphones for life services such as online transactions, financial transactions, and life management, and search for information such as news and the location of stores and services [18].

The use of apps appears to affect not only mothers’ childrearing and home situations but also their psychological well-being. Studies have shown that the support mothers experience through convenient mobile phone apps is helpful in ameliorating post-partum depression and childrearing difficulties [19,20]. Furthermore, for those with higher self-efficacy, mobile phones have more positive effects on users’ attitudes [21,22]. Choi and Koo [9] showed that smartphones can serve as a safe haven in an environment where emotions cannot be freely expressed and as a tool to accept frustration when irrational desires or expectations in married life have not been met. These findings demonstrate that smartphones not only support mothers’ childrearing efforts but also affect their emotions.

However, thus far, no study has thoroughly explored the subjective characteristics of smartphone use with respect to the meanings and values it has for mothers. For mothers of young children to use smartphones prudently, they must develop an in-depth awareness and understanding of smartphones’ effects. Therefore, this study analyzed mothers’ perceptions of smartphone use to determine its role in parenting. We proposed the following research questions:

**RQ1.**  *In terms of their subjective perceptions, what are the types of smartphone users among mothers raising young children?*

**RQ2.**  *What are the characteristics of these types of smartphone users?*

## 2. Materials and Methods

The Q methodology was introduced by psychologist and physicist William Stephenson in 1935, and it groups people with similar characteristics such as attitudes toward and behaviors relating to related topics [23,24]. The main purpose of this research method is to explore people’s perceptions and opinions [25]. This study utilized the Q methodology, which explores the subjectivity of a given issue—in this study, mothers’ perceptions of smartphone apps use in raising young children—by quantifying the subjective tendencies and values of individuals and categorizing them [26,27]. When conducting research with Q methodology, it is important to select appropriate statements on relevant topics that reflect subjectivity and to determine how much the study participants agree with and disagree with those statements [28].

**Figure 1:**
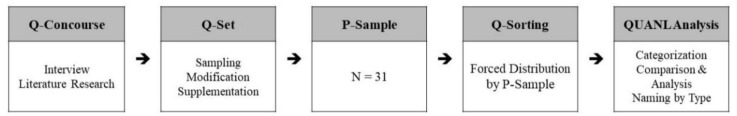
Research process.

Figure 1 depicts the research process. The process begins with the creation of a Q- concourse. The Q-set is selected from the Q concourse to be used for Q sorting. The selected P samples participate in Q sorting, and the data collected from the sort are analyzed and interpreted using the QUANL program.

Q-concourse refers to the range of items or statements that exist on a specific topic. To this end, interviews, literature reviews, media reports, and pictures can be used in various ways depending on research aims [25]. Q-set is also called Q-sort pack or Q-sample and refers to representative items or statements selected from the Q-concourse [29]. P sample refers to people who participate in Q-sorting, which is the process of classifying items or statements according to the degree to which participants agree and disagree with them [30]. The data and analysis process refers to the process of coding data (Q-sorting), entering it into a QUNAL program, and analyzing and interpreting the results.

### 2.1. Q-Concourse and Q-Set

In this study, the Q-concourse includes the entire set of perceptions of smartphones among mothers with infants, and the Q-set includes representative and comprehensive statements extracted from the Q-concourse. We conducted interviews and literature reviews to collect the Q-concourse. First, we conducted face-to-face interviews with four mothers and virtual interviews with two mothers. Each interview lasted approximately 30–60 min. The interviews were related to parenting and included questions such as “What do you think about using a smartphone?”, “When do you use a smartphone?”, and “What does a smartphone mean to you?” Through this process, 80 statements that revealed participants’ conceptions of, perceptions of, attitudes toward, and usage of smartphones were extracted. Further, 61 statements were extracted from literature on smartphones [31,32,33,34,35]. Thus, the Q-concourse comprised a total of 141 statements. The process of collecting the Q-concourse proceeds until the content of each statement is repeated and each statement is judged to contain all possible content [28]. The collected Q-concourse of 141 was divided into five domains: mother and housewife roles, excessive addictiveness, convenience and functions, communication roles, and a personal domain for selecting the statements in a comprehensive and representative manner.

For selection of the Q-set from the Q-concourse, common meanings or valuable elements were extracted while reading the Q-concourse repeatedly; overlapping content was excluded, and the remaining content was integrated by subject and then categorized. The selected 40 statements spanned five categories, with 8 statements on mother and housewife roles, 5 on excessive addictiveness, 8 on convenience and functions, 8 on communication roles, and 11 on the personal domain. We sought advice from an expert on the discriminatory power and validity of the categories’ content (i.e., whether participants were accurately represented by the categories). To derive the Q-samples, we selected statements with various and representative characteristics in relation to participants from the categories. Using the 40 extracted statements, we conducted a preliminary survey with three mothers to revise items whose meaning was not communicated clearly. The Appendix A contains the 40 statements in the Q-set.

### 2.2. Participants (P Sample)

P samples refer to those who participate in the Q-sorting of the Q-set. In the Q methodology, the selection of P samples is deemed sufficient if participants are evenly distributed with respect to the purpose of the study and range from 10–100 individuals [26,36]. This study’s purposive sampling method selected mothers with young children (*n* = 31; age range = 20 s–40 s) who use smartphones. The study’s purpose was clearly explained to participants (P samples), as was the anonymity of their responses. Participants could withdraw from the study at any time, and they all provided written informed consent to participate.

### 2.3. Q-Sorting and Data Analysis

According to Brown [37], Q-sorting is a procedure in which participants (P-samples) are instructed to rank Q-statements (Q-set) in order of importance under specified conditions. We asked participants to review the 40 Q-statements and rank them on an 11-point scale from the most agreeable (+5) to the most disagreeable (−5). Next, participants classified the statements by placing them on a predetermined distribution pattern called a Q-sort table. Participants then wrote in detail about the two subjective statements at each end of their scale.

We analyzed the results from Q-sorting using the Q principal components factor analysis function within the QUANL program. We categorized the resulting data (z scores) from the factor analysis into types, extracting only those with an eigenvalue of ±1.000 or more. We performed further analysis on participants’ written explanations about their most agreeable and disagreeable statements for in-depth interpretation.

## 3. Results

### 3.1. Types and Characteristics of Smartphone Users Based on Mothers’ Perceptions

The analysis yielded four types of smartphone users based on mothers’ perceptions. The four types accounted for 43.82% of the total variance. Table 1 displays the eigenvalues and variance for each type.

Factor weights indicate how much each type showed a given characteristic. Table 2 shows the factor weights of the individual P samples.

#### 3.1.1. Type 1: “Multi-Adapter” Who Actively Utilizes Functions

The first type of smartphone user was named “multi-adapters who actively utilize functions” because these individuals actively use apps that enable communication and provide information. As shown in Table 3, the statements with which participants strongly agreed were “I feel that it is important to teach children healthy habits for using smartphones” (*z* = 2.20), “I feel that I have an advantage if I am able to effectively use the apps on my smartphone” (*z* = 1.22), and “I feel that smartphones are helpful in providing easy access to websites where mothers share information and their experiences regarding childrearing” (*z* = 1.08). The statements with which Type 1 individuals disagreed the most were “I feel that my smartphone allows me to indirectly boast as much as I want to without having to worry about what others will think of me” (*z* = −1.81) and “I feel that I have no choice but to use my smartphone when I want to quickly resolve my child’s tantrums” (*z* = −1.80).

P18, who belonged to the first type, stated that “The hours of smartphone use should fit the age of the child, and searching for information together with children and sufficiently utilizing the information within the given time are helpful and important for the development of children”. Additionally, P24 remarked, “Using mobile phones and information technology (IT), many advantages such as [the] internet protocol camera, video calls, and positioning using a GPS can be utilized”.

These individuals leverage the strengths of smartphones in step with smartphones’ development and do so together with their children, carrying out smartphone education in the process. However, they tend to perceive indiscriminate use of smartphones, using smartphones for releasing personal emotions, and using smartphones for self-display through social networking services (SNS) negatively.

#### 3.1.2. Type 2: “Chaos Dilemma Type” Who Knows the Disadvantages of Smartphones but Cannot Help but Use Them

The second type of smartphone user was named the “chaos dilemma type,” as these individuals tend to experience internal conflicts because the overwhelming information from smartphones begins to affect their own lifestyles and views on childrearing. As shown in Table 4, the statements with which they strongly agreed were “I feel uncertain about my perspective and values regarding childrearing due to constant exposure to social media posts about raising children” (*z* = 1.43), “I feel that I am making more impulse purchases because it is quick and easy to shop as well as send gifts via apps” (*z* = 1.38), and “I feel that smartphones are helpful in providing easy access to websites where mothers share information and their experiences regarding childrearing” (*z* = 1.16). The statements with which Type 2 individuals strongly disagreed were “I feel physically and mentally drained from smartphone overuse” (*z* = −1.53), and “I feel that the pros of using a smartphone outweigh the cons” (*z* = −2.47).

P06, who belonged to the second type, stated that “Shopping using a smartphone saves time, is convenient, and enables seeing a variety of information at a glance. Therefore, I often and easily buy mobile gifts, so I give gifts to others more than necessary”. P20 remarked, “I seem to experience regret after buying too many things because I can easily purchase things that are not easily available around me using mobile shopping”. Additionally, P20 stated, “As a user of the ‘Mom Café’, I worry more and feel difficulties in making decisions, as I try to refer to all the objective opinions of mothers in similar situations around me rather than raising my children only with subjective educational views and values”.

These users utilize smartphones in various functional areas such as shopping and information acquisition to assist in their daily lives. They perceive smartphones as allowing them to share opinions about the difficulties of parenting with experienced people and learn parenting methods. However, they experience the dilemma of knowing that excessive use of smartphones is inappropriate because their views of parenting are shaken and their consumption and expenditures increase due to excessive information, yet they are unable to stop using smartphones. Nonetheless, these individuals do not believe that excessive use of smartphones causes mental and physical problems.

#### 3.1.3. Type 3: “Time-Killer Dependent Type” Who Relieves Temporary Stress and Anxiety

The third type of smartphone user exhibits the characteristic of dependence. They feel guilty because they believe that they are using smartphones excessively to solve their emotional difficulties, but they still use the devices. Therefore, Type 3 was named the “time-killer dependent type” who use smartphones to relieve temporary stress and anxiety. As shown in Table 5, the statements with which these individuals strongly agreed were “I feel that I consciously try to control how much time I spend on my smartphone because I am afraid that my child might model my smartphone overuse behavior” (*z* = 1.88), “I tend to overuse my smartphone when I feel anxious or depressed” (*z* = 1.29), “I feel guilty whenever I use my smartphone for a long time because I feel that I have neglected my parenting responsibilities” (*z* = 1.27), and “I feel that using smartphones is a great way to kill time and have fun” (*z* = 1.17). The statements with which they disagreed the most were “I feel that I tend to judge people before I even meet them based on what I see on their social media profile” (*z* = −1.90) and “I feel that my smartphone allows me to indirectly boast as much as I want to without having to worry about what others will think of me” (*z* = −1.83).

P08, who belonged to the third type, stated that “Even when I am spending my time alone, I don’t feel like I have taken a break because I am doing online shopping and using SNS”. Additionally, P20 remarked, “The time I spend habitually looking at my smartphone continues to increase whenever I have free time, but I do not feel a fundamental sense of relief. Therefore, I feel like I am addicted”. P12 also stated, “Even on days when my body is tired, when I engage in fun activities such as smartphone shopping or SNS rather than taking a break, I forget everything, at least for that period of time”.

This type uses their smartphone whenever they have free time and unconsciously uses smartphones even when they need physical rest. They use smartphones more when they are in mentally and physically exhausting situations. Additionally, they use smartphones as a psychological defense to temporarily forget uncomfortable emotions, with the intention to find comfort or pleasure. However, they seem conscious of their dependence on smartphones and that using them in such a way is only a temporary solution.

#### 3.1.4. Type 4: “Self-Development-Focused User Type” Who Seeks Positive Changes

The fourth type of smartphone user pursues positive changes while finding various information, trying recommended things, and enjoying recuperation through communication and personal time. Therefore, they were named the “self-development-focused user type”. As shown in Table 6, the statements with which they strongly agreed were “I feel that using smartphones is a great way to kill time and have fun” (*z* = 1.47), “I feel that smartphones are necessary to enjoy my free time after I finish my household chores and put the children to bed” (*z* = 1.22), and “I feel that having access to diverse applications on my smartphone allows me to expand my knowledge and experiences in life” (*z* = 1.14). The statements with which Type 4 individuals disagreed the most were “I feel that I have no choice but to use my smartphone when I want to quickly resolve my child’s tantrums” (*z* = −1.90), “I tend to overuse my smartphone when I feel anxious or depressed” (*z* = −1.72), and “I feel that I tend to compare myself to others whenever I look at social media on my smartphone, which leads me to have low self-esteem” (*z* = −1.28). P30, who belonged to the fourth type, stated that “After finishing the housework and putting the children to sleep, I enjoy lying down comfortably and searching for various information”. P14 remarked, “While I talk with my acquaintances, I feel like I want to follow their good methods”.

These individuals make appropriate use of their smartphones’ functions, receive quality information, and pursue positive changes. They tend to seek rest and enjoyment and experience comfort and rejuvenation through SNS communication. However, they do not agree with using smartphones regularly as a tool to appease their irritable children. Additionally, they seek development such as communication and information exchange with others and resolve difficulties through relationships, and they do not use smartphones more often when depressed or anxious.

## 4. Discussion

Smartphone use is associated with quality of life [38]. Accordingly, this study examined how mothers with young children perceive the role of smartphones in their lives.

Type 1 mothers (“multi-adapters”) have become proficient with smartphones and IT as members of the millennial generation; that is, they show the characteristics of “Homo digipiens”, which are adapted to digital civilization and thus are familiar with various wearable devices. According to Kang [39], mothers actively use voice functions equipped with artificial intelligence, health alarms, and automatic location tracking functions, and it can be seen from the results of this research that Type 1 has the ability to actively utilize these functions. They consider the advantages and disadvantages of smartphones before using them and make efforts to teach their children healthy smartphone use habits. Our findings accord with those of Kim [40], which indicated that married women consume rich media content in various situations due to living in the age of smartphones. However, Type 1 users reject behaviors such as using smartphones as tools to meet emotional needs or compulsively relying on smartphone functions. 

Type 2 mothers (“chaos dilemma type”) seek the opinions of experienced people in various ways and frequently use SNS to find convenient and accurate information. However, due to excessive information and notifications, they experience confusion in their views on childrearing, and their lifestyles indicate that they have difficulties in coping with this confusion. Despite this, various uses of smartphones allow them to reduce their trial-and-error learning while raising children and help them share information with other mothers. In this respect, the second type of mother exhibits characteristics consistent with Lee’s [41] findings, which indicated that mobile shopping using smartphones is a positive experience in terms of the diversity of product information, ease of searching, and ease of use. However, it implies the possibility of an impulsive overconsumption tendency. This can be seen as a dysfunctional social consequence of informatization.

Type 3 mothers (“time-killer dependent type”) use smartphones for pleasure. This is consistent with the finding that using smartphone apps such as Facebook significantly affects one’s desire for entertainment [42]. Additionally, because they use smartphones to escape negative emotions or stressful situations (rather than for a concrete goal), they usually use them to watch simple content, surf the web, and look at SNS. These mothers also use smartphones to eliminate or reduce anxiety and depression. Kang and Shin [43] argued that loneliness is a decisive factor in smartphone dependence. Rather than looking for active, communicative alternatives, these mothers use one-sided methods, finding solutions or comfort from indirectly looking into others’ lives, in which smartphones play a temporary relieving role. In fact, a study by Choi and Koo [9] found that suppressed emotions, beliefs, and desires are inherent in the lives of married women who use social media excessively. Thus, it is evident that dependence on smartphones has psychological consequences for mothers. Recently, a mindfulness app has been used to ameliorate these psychological difficulties [44], and mothers who use postpartum mobile support applications have been found to have reduced anxiety and depression [45]. However, mothers such as those in Type 3 will not be able to receive appropriate benefits if they do not have information about apps that can effectively provide psychological support. Therefore, accessibility to these apps should be improved to help mothers achieve sustainable well-being.

Type 4 (“self-development-focused user type”) mothers attempt to create positive experiences for themselves and pursue positive changes or personal growth by using smartphones. These mothers actively engage in productive activities on SNS. They expand their interpersonal relationships and resolve life challenges through various SNS. Type 4 mothers show similarities to Type 1 mothers in that they use smartphone functions flexibly and leverage the advantages of information. Nevertheless, there is a meaningful difference between the two types: whereas the use of functions and information is more important for Type 1 mothers, personal changes and developmental experiences are more important for Type 4 mothers.

This study is meaningful in that it explored the subjective meaning and value of smartphones among mothers with young children. Based on the present findings, we make the following suggestions. 

First, for Type 1 mothers, who use smartphones’ functional and informational components in a rational and useful manner, apps facilitating safe and healthy communication that is necessary for this group to achieve balanced lives should be developed and supplied. Additionally, there should be efforts to enable efficient utilization of practical and useful information on childrearing as well as to provide information on childcare and educational facilities.

Second, Type 2 mothers may experience disruptions in their emotional and psychological balance due to conflicting views on childrearing brought about by information they acquire on smartphones. They tend to indiscriminately accept information provided by smartphones, and they require a method of clear and objective evaluation of the quality of information. Thus, a system that can disseminate high-quality information to this group should be established (e.g., ranking the quality of information).

Third, Type 3 mothers use smartphones to relieve stress, which suggests that they are very dependent on smartphones. To maximize their potential to reap the benefits of smartphones for psychological comfort, it is necessary to develop a counseling app for this group.

Fourth, Type 4 mothers are active users of smartphones for self-development purposes. It is thus necessary to organize social opportunities for healthy expression of their active energy and change-seeking propensities, as well as to devise systems that promote not only online but also offline connections.

## 5. Conclusions

Our analysis yielded four types of smartphone-using mothers, who were categorized according to their respective characteristics. Uses of smartphones that fit these different profiles should thus be pursued.

## 6. Limitations

This study explored how the subjective perceptions of smartphone app use are grouped for mothers of young children using Q methodology. Through this method, the subjective and psychological structure of each group was revealed, but the Q methodology is not intended to be generalizable. Therefore, there are limitations that need to be overcome by analyzing the results of future quantitative research on the types of mothers that are raising young children and the demographic backgrounds of mothers belonging to each type. In addition, in future research, it is necessary to analyze in depth how each type of mother uses and maintains smartphone apps through interviews.

## Figures and Tables

**Table 1:** Eigenvalues and variance for the four types of smartphone users.

Content/Type	I	II	III	IV
Eigenvalue	6.9194	2.6730	2.1034	1.8882
Total Variance	0.2232	0.0862	0.0679	0.0609
Cumulative	0.2232	0.3094	0.3773	0.4382
Solution Variance	0.5094	0.1968	0.1548	0.1390
Cumulative	0.5094	0.7062	0.8610	1.0000

**Table 2:** Sociodemographic characteristics and factor weights by type.

	P Sample	Age	Number of Children	Employed	Factor Weight
Type 1 (*n* = 8)	04	39	1	No	2.0118
	18	40	2	Yes	1.8313
	28	38	1	No	1.5010
	16	39	1	Yes	1.1184
	05	35	2	No	1.1695
	07	32	3	No	0.7832
	24	43	1	Yes	0.5607
	23	35	2	No	0.3050
Type 2 (*n* = 8)	02	38	1	No	1.0795
	25	36	2	Yes	0.7521
	09	31	2	Yes	0.6799
	21	40	2	Yes	0.6558
	22	42	2	Yes	0.6426
	20	37	1	No	0.5034
	27	35	3	No	0.5018
	06	33	2	No	0.3648
Type 3 (*n* = 11)	11	35	2	Yes	1.4353
	31	43	2	Yes	1.3750
	08	40	2	No	1.2968
	13	35	2	No	0.8138
	29	42	2	Yes	0.7237
	17	38	1	Yes	0.6408
	01	30	3	Yes	0.6366
	26	29	2	No	0.5504
	12	39	3	Yes	0.5087
	19	37	1	Yes	0.4345
	15	29	1	No	0.2983
Type 4 (*n* = 4)	14	35	2	No	1.1376
	10	22	1	Yes	0.9269
	03	30	2	No	0.7838
	30	32	2	No	0.7122

**Table 3:** Statements and standard scores for Type 1 users (≥±1.00).

No.	Statement	Standard Score
15	I feel that it is important to teach children healthy habits for using smartphones.	2.20
16	I feel that smartphone addiction problems have more to do with problems in our society than personal issues.	1.91
33	I feel that I consciously try to control how much time I spend on my smartphone because I am afraid that my child might model my smartphone overuse behavior.	1.76
24	I feel worried sometimes that there might be a miscommunication or misunderstanding after I answer texts in a rush.	1.51
3	I feel that I have an advantage if I am able to effectively use the apps on my smartphone.	1.22
5	I feel that smartphones are helpful in providing easy access to websites where mothers share information and their experiences regarding childrearing.	1.08
2	I feel that smartphones are a good way to keep my children entertained while I am busy doing other things.	1.04
30	I feel that communicating through smartphones is a better way to resolve family conflicts.	−1.12
7	I feel that using my smartphone helps me release my stress.	−1.19
25	I am very concerned about the self-image I portray on social media.	−1.24
11	I feel that my smartphone’s convenience in terms of size and function allows me to enjoy using it while taking care of my children.	−1.28
9	I feel that the pros of using a smartphone outweigh the cons.	−1.33
4	I feel that I tend to compare myself to others whenever I look at social media on my smartphone, which leads me to have low self-esteem.	−1.37
22	I feel that I have no choice but to use my smartphone when I want to quickly resolve my child’s tantrums.	−1.80
23	I feel that my smartphone allows me to indirectly boast as much as I want to without having to worry about what others will think of me.	−1.81

**Table 4:** Statements and standard scores for Type 2 (≥±1.00).

No.	Statement	Standard Score
32	I feel uncertain about my perspective and values regarding childrearing due to constant exposure to social media posts about raising children.	1.43
28	I feel that I am making more impulse purchases because it is quick and easy to shop as well as send gifts via apps.	1.38
15	I feel that it is important to teach children healthy habits for using smartphones.	1.18
5	I feel that smartphones are helpful in providing easy access to websites where mothers share information and their experiences regarding childrearing.	1.16
22	I feel that I have no choice but to use my smartphone when I want to quickly resolve my child’s tantrums.	1.13
27	I feel like my smartphone is addicting because of its easy access to information and continual notifications for text messages.	1.00
10	I feel that using my smartphone to search for information related to my daily needs is an extension of my household chores.	−1.01
23	I feel that my smartphone allows me to indirectly boast as much as I want to without having to worry about what others will think of me.	−1.08
18	I feel burdened because of the pressure of having to respond quickly to people using my smartphone.	−1.29
4	I feel that I tend to compare myself to others whenever I look at social media on my smartphone, which leads me to have low self-esteem.	−1.30
25	I am very concerned about the self-image I portray on social media.	−1.47
14	I feel physically and mentally drained from smartphone overuse.	−1.53
7	I feel that using my smartphone helps me release my stress.	−1.86
9	I feel that the pros of using a smartphone outweigh the cons.	−2.47

**Table 5:** Statements and standard scores for Type 3 (≥±1.00).

No.	Statement	Standard Score
15	I feel that it is important to teach children healthy habits for using smartphones.	2.16
33	I feel that I consciously try to control how much time I spend on my smartphone because I am afraid that my child might model my smartphone overuse behavior.	1.88
5	I feel that smartphones are helpful in providing easy access to websites where mothers share information and their experiences regarding childrearing.	1.51
38	I tend to overuse my smartphone when I feel anxious or depressed.	1.29
8	I feel guilty whenever I use my smartphone for a long time because I feel that I have neglected my parenting responsibilities.	1.27
6	I feel that using smartphones is a great way to kill time and have fun.	1.17
21	I feel that my smartphone distracts me from doing the things I need to do.	1.14
20	I feel that smartphones are necessary to enjoy my free time after I finish my household chores and put the children to bed.	1.04
19	I feel that I have more of a tendency and desire to flaunt and pretend to have a luxurious life in front of others since I started using social media through my smartphone.	−1.16
37	I feel that my smartphone allows me to meet a lot of people, especially during a period in which I feel restricted in going out to socialize due to my responsibilities as a mother.	−1.18
25	I am very concerned about the self-image I portray on social media.	−1.25
26	I feel that it would be hard to control my child’s smartphone use if my child had access to a smartphone too early.	−1.76
23	I feel that my smartphone allows me to indirectly boast as much as I want to without having to worry about what others will think of me.	−1.83
40	I feel that I can participate in community affairs through my smartphone.	−1.87
39	I feel that I tend to judge people before I even meet them based on what I see on their social media profile.	−1.90

**Table 6:** Statements and standard scores for Type 4 (≥±1.00).

No.	Statement	Standard Score
2	I feel that smartphones are a good way to keep my children entertained while I am busy doing other things.	1.61
6	I feel that using smartphones is a great way to kill time and have fun.	1.47
18	I feel burdened because of the pressure of having to respond quickly to people using my smartphone.	1.34
17	I feel overwhelmed because there is too much information available through my smartphone.	1.27
20	I feel that smartphones are necessary to enjoy my free time after I finish my household chores and put the children to bed.	1.22
27	I feel like my smartphone is addicting because of its easy access to information and continual notifications for text messages.	1.20
36	I feel that being able to share experiences with and talk to people I can relate to through my smartphone helps me overcome the difficulties I face while raising my children.	1.18
34	I feel that having access to diverse applications on my smartphone allows me to expend my knowledge and experiences in life.	1.14
5	I feel that smartphones are helpful in providing easy access to websites where mothers share information and their experiences regarding childrearing.	1.09
35	I feel that my smartphone allows me to easily and efficiently take care of my daily errands.	1.07
16	I feel that smartphone addiction problems have more to do with problems in our society than personal issues.	−1.00
7	I feel that using my smartphone helps me release my stress.	−1.05
31	I feel that using my smartphone (e.g., to listen to music or look at my social media comments) makes me feel more comforted than meeting up with a close friend.	−1.17
30	I feel that communicating through smartphones is a better way to resolve family conflicts.	−1.21
4	I feel that I tend to compare myself to others whenever I look at social media on my smartphone, which leads me to have low self-esteem.	−1.28
40	I feel that I can participate in community affairs through my smartphone.	−1.36
38	I tend to overuse my smartphone when I feel anxious or depressed.	−1.72
22	I feel that I have no choice but to use my smartphone when I want to quickly resolve my child’s tantrums.	−1.90

## Data Availability

Data from the study are available upon request.

## Appendix A

**No.**	**Q-Set (Q-Statements)**	**Domains**
Q1	I feel that I can experience diverse cultures because I can easily buy foreign products from websites using my smartphone.	Mother and housewife roles
Q2	I feel that smartphones are a good way to keep my children entertained while I am busy doing other things.	Mother and housewife roles
Q3	I feel that I have an advantage if I am able to effectively use the apps on my smartphone.	Convenience and functions
Q4	I feel that I tend to compare myself to others whenever I look at social media on my smartphone, which leads me to have low self-esteem.	Personal
Q5	I feel that smartphones are helpful in providing easy access to websites where mothers share information and their experiences regarding childrearing.	Mother and housewife roles
Q6	I feel that using smartphones is a great way to kill time and have fun.	Personal
Q7	I feel that using my smartphone helps me release my stress.	Personal
Q8	I feel guilty whenever I use my smartphone for a long time because I feel that I have neglected my parenting responsibilities.	Excessive addictiveness
Q9	I feel that the pros of using a smartphone outweigh the cons.	Convenience and functions
Q10	I feel that using my smartphone to search for information related to my daily needs is an extension of my household chores.	Mother and housewife roles
Q11	I feel that my smartphone’s convenience in terms of size and function allows me to enjoy using it while taking care of my children.	Convenience and functions
Q12	I feel that smartphone apps are especially useful when I need to contact and maintain relationships with those who I want to avoid.	Communication roles
Q13	I feel that smartphones are necessary for obtaining information to learn about better childrearing practices.	Mother and housewife roles
Q14	I feel physically and mentally drained from smartphone overuse.	Excessive addictiveness
Q15	I feel that it is important to teach children healthy habits for using smartphones.	Mother and housewife roles
Q16	I feel that smartphone addiction problems have more to do with problems in our society than personal issues.	Excessive addictiveness
Q17	I feel overwhelmed because there is too much information available through my smartphone.	Convenience and functions
Q18	I feel burdened because of the pressure of having to respond quickly to people using my smartphone.	Personal
Q19	I feel that I have more of a tendency and desire to flaunt and pretend to have a luxurious life in front of others since I started using social media through my smartphone.	Communication roles
Q20	I feel that smartphones are necessary to enjoy my free time after I finish my household chores and put the children to bed.	Personal
Q21	I feel that my smartphone distracts me from doing the things I need to do.	Personal
Q22	I feel that I have no choice but to use my smartphone when I want to quickly resolve my child’s tantrums.	Mother and housewife roles
Q23	I feel that my smartphone allows me to indirectly boast as much as I want to without having to worry about what others will think of me.	Communication roles
Q24	I feel worried sometimes that there might be a miscommunication or misunderstanding after I answer texts in a rush.	Personal
Q25	I am very concerned about the self-image I portray on social media.	Communication roles
Q26	I feel that it would be hard to control my child’s smartphone use if my child had access to a smartphone too early.	Mother and housewife roles
Q27	I feel like my smartphone is addicting because of its easy access to information and continual notifications for text messages.	Personal
Q28	I feel that I am making more impulse purchases because it is quick and easy to shop as well as send gifts via apps.	Convenience and functions
Q29	I feel a sense of achievement thanks to my smartphone’s easy access to YouTube, where I can easily learn housekeeping tips.	Convenience and functions
Q30	I feel that communicating through smartphones is a better way to resolve family conflicts.	Communication roles
Q31	I feel that using my smartphone (e.g., to listen to music or look at my social media comments) makes me feel more comforted than meeting up with a close friend.	Personal
Q32	I feel uncertain about my perspective and values regarding childrearing due to constant exposure to social media posts about raising children.	Personal
Q33	I feel that I consciously try to control how much time I spend on my smartphone because I am afraid that my child might model my smartphone overuse behavior.	Excessive addictiveness
Q34	I feel that having access to diverse applications on my smartphone allows me to expend my knowledge and experiences in life.	Convenience and functions
Q35	I feel that my smartphone allows me to easily and efficiently take care of my daily errands.	Convenience and functions
Q36	I feel that being able to share experiences with and talk to people I can relate to through my smartphone helps me overcome the difficulties I face while raising my children.	Communication roles
Q37	I feel that my smartphone allows me to meet a lot of people, especially during a period in which I feel restricted in going out to socialize due to my responsibilities as a mother.	Communication roles
Q38	I tend to overuse my smartphone when I feel anxious or depressed.	Excessive addictiveness
Q39	I feel that I tend to judge people before I even meet them based on what I see on their social media profile.	Communication roles
Q40	I feel that I can participate in community affairs through my smartphone.	Personal
